# A non-interventional, observational study of a fixed combination of pepsin and amino acid hydrochloride in patients with functional dyspepsia

**DOI:** 10.1186/s12876-017-0675-9

**Published:** 2017-11-25

**Authors:** Kristin Forssmann, Larissa Meier, Bernhard Uehleke, Cornelia Breuer, Rainer Stange

**Affiliations:** 1 Nordmark Arzneimittel GmbH & Co. KG, Pinnauallee 4, D-25436 Uetersen, Germany; 2 Charité - University Medicine Berlin and Immanuel Krankenhaus, Koenigstrasse 63, D-14109 Berlin, Germany

**Keywords:** Functional dyspepsia, Observational trial, Non-interventional trial, Natural remedy, Enzyme therapy, Pepsin

## Abstract

**Background:**

Functional dyspepsia (FD) is a gastrointestinal disorder characterized by recurrent and diverse symptoms and pathophysiology that remains unexplained following routine clinical investigation. Enzynorm®*f* is a pharmaceutical preparation comprising fixed amounts of pepsin of biological origin and organically bound acid in the form of amino acid hydrochloride. It is traditionally used as a mild agent to support gastric function and to stimulate the stomach’s proteolytic activities in FD.

**Methods:**

In a non-interventional, observational, post-marketing surveillance study, patients with an established diagnosis of FD were treated with a fixed combination of pepsin and amino acid hydrochloride taken as tablets three times daily for 6 weeks. The primary objective of this study was to assess the change in symptoms using the validated Gastrointestinal Symptom Score (GIS©). Secondary objectives included patients’ assessment of their gastrointestinal symptoms as well as treatment safety and tolerability.

**Results:**

A total of 97 patients (mean age 58.4 ± 13.9 years; 63.2% females) were included in the study, with 72 data having GIS© score data at baseline and at 6 weeks, and 34 also at 3 weeks. The overall GIS© sum score decreased by 4.1 (*p* < 0.0001) from 11.6 (±4.8) at baseline to 7.4 (± 4.6) reflecting an improvement of clinical symptomatology after 6 weeks of treatment. In a subgroup of 70 patients who had FD meeting the Rome III criteria a GIS© score reduction of ≥50% was observed after 3 weeks treatment in 24% and in 30.8% after 6 weeks. Adverse events were mostly gastrointestinal in nature and consistent with the underlying disease; no unexpected adverse reactions were reported. Twenty-seven patients discontinued the study, mostly because of gastrointestinal symptoms.

**Conclusion:**

The results of this study support the efficacy of a fixed combination of pepsin and amino acid hydrochloride for the treatment of patients with FD and also suggest good to moderate treatment tolerability. These findings should be further explored in a randomised, placebo-controlled clinical trial.

**Clinical trial registration:**

This study has been retrospectively registered in the ClinicalTrials.gov registry, trial identifier NCT03076411.

## Background

Functional dyspepsia (FD) is a gastrointestinal disorder characterized by recurrent and diverse symptomatology and pathophysiology [1–3]. According to the Rome III criteria, symptoms include, but are not limited to postprandial fullness, early satiation, epigastric pain, and epigastric burning that remain unexplained following routine clinical investigation [4]. A number of treatment approaches are available, including proton pump inhibitors, eradication of *Helicobacter pylori*, prokinetic agents, and fundus-relaxing drugs [1, 3]; however, management of FD can be challenging mainly due to frequent relapse [3].

Functional dyspepsia is one of the most common functional disorders affecting the adult population in Western countries with an estimated prevalence between 20 and 25% [1], while there are estimates for global prevalence as high as 45% [5]. Functional dyspepsia appears to occur more frequently in females than in males [5]. The average age of the affected patient population seems to be about 45 years, and, although estimates vary, it is clearly more prevalent in those older than 40 years of age [5]. Although not life-threatening nor damaging to any organ, FD is a burden for patients and is associated with excess comorbidity [6, 7]. It also leads to high costs due to diagnostics and medication, and can also cause excess work absenteeism and decreased productivity [6, 7].

Enzynorm®*f* (manufactured by Nordmark Arzneimittel GmbH & Co. KG, Uetersen, Germany) is a pharmaceutical preparation comprising fixed amounts of pepsin of biological origin and organically bound acid in the form of amino acid hydrochloride [8]. It is traditionally used as a mild agent for support of gastric function and to stimulate the stomach’s proteolytic activities in FD [8, 9] and has been marketed in Germany for more than 80 years [10]. The rationale for its possible effects is that pepsin as, a proteolytic enzyme has a key role in the initial hydrolysis of ingested proteins into smaller peptides in the stomach and therefore additional pepsin and acidic components should support digestion [9]. Despite its long history of usage in patients with FD, however, there is a lack of scientific data on the use of Enzynorm®*f* or other digestive enzyme preparations that are available for the treatment of FD. Only some small-scale studies with pancreatic enzymes, typically showing proteolytic, lipolytic and carboxylic activities, for treating disorders including FD have been published [11, 12]. In the absence of any clinical study data on the use of Enzynorm®*f* for the treatment of FD, we have conducted an observational study to evaluate the effects of this fixed combination of pepsin and amino acid hydrochloride on the course of symptoms and tolerability during 6 weeks’ treatment.

## Methods

### Study design and objectives

This was a non-interventional, observational, post-marketing surveillance study. The primary objective was to assess the change of symptoms following treatment with a fixed combination of pepsin and amino acid hydrochloride when used as daily standard therapy over a period of approximately 6 weeks. Secondary objectives were patients’ assessments of the frequency and intensity of their gastrointestinal symptoms and tolerability of treatment.

The study was performed in the outpatient clinic of the department for Natural Medicine of Charité at Immanuel Hospital, University Medicine Berlin, Germany, between January 2012 and December 2013. The study was brought to patients’ attention by means of advertisements in the local press and on the hospital’s website.

Patients were examined at baseline, after 3 and after 6 weeks of treatment for assessment of symptoms and tolerability. In addition, they were contacted by telephone approximately 1 week after they had started the study.

The study was performed in compliance with Good Clinical Practice (according to GCP-V, 2004) and all applicable laws and regulations. Written informed consent was obtained from all patients. The study design was approved by the Ethics Committee of Charité, University Medicine Berlin, Germany, prior to the study. The study sponsor was Nordmark Arzneimittel GmbH & Co. KG, Uetersen, Germany.

### Patients

The study included male and female patients between 18 and 75 years of age with a diagnosis of acute or chronic FD, as verified by their medical history. To be included, patients were required to have had at least 2 of the following gastrointestinal symptoms present during at least the previous 4 weeks with at least moderate severity. The latter was defined as moderately severe and moderately troublesome on at least 5 days in the prior 2 weeks: bad breath, burping, nausea, heartburn, feeling of fullness, epigastric pain (Table 1). Patients were also required to be evaluated according to the Rome III criteria for FD [13, 14] prior to inclusion (the study was conducted prior to the introduction of the Rome IV criteria [4]).

**Table 1:** Study inclusion and exclusion criteria

Demographic inclusion criteria
• Males and females
• Age ≥ 18 years to ≤75 years of age
Clinical inclusion criteria
• Acute or chronic functional dyspepsia verifed by medical history
• ≥ 2 of the following gastrointestinal symptoms at moderate severity^a^within prior 4 weeks:
○ Bad breath, burping, nausea, heartburn, feeling of fullness, epigastric pain
• Undergo evaluation according to the Rome III criteria for FD [13, 14]^b^
Exclusion criteria
• Contraindications to study treatment (e.g., Known allergies to active ingredients or excipients)
• Presence of alarm symptoms
• Significant accompanying gastrointestinal or other disease
• Pregnancy or nursing of infant(s)
• Patients with changes to medication within prior 6 weeks
• Previous intake of pepsin and amino acid hydrochloride

^a^Defined as moderately severe and moderately troublesome on ≥5 days in the prior 2 weeks

^b^The study was conducted prior to introduction of the Rome IV criteria [4]

In addition to the study treatment, patients continued their usual therapy for FD during the study. Patients with contraindications for the study treatment, such as known allergies against the active ingredients or any of the excipients, were excluded from the study. Patients showing alarm symptoms, patients with significant accompanying gastrointestinal or other diseases, pregnant or nursing women were not considered eligible, and neither were patients with a change in medication over the last 6 weeks or previous intake of pepsin and amino acid hydrochloride.

### Treatment

The active constituents of a single Enzynorm®*f* tablet are 225–250 mg dry extract of porcine gastric mucosa with proteinases plus 250 mg amino acid hydrochloride from bovine albumin hydrolysate [8]. Commonly, tablets are to be swallowed unbroken three times daily before meals with some liquid. Dosing was left to the judgement of the physician following the scheme used in patients’ standard care and in accordance with the usage information that means 3 × 1 as usual, 3 × 2 as augmented and up to 20 tablets daily as maximum dosage [8].

### Outcome parameters

The primary outcome parameter was the change from baseline in FD symptoms as evaluated with the Gastrointestinal Symptom Score© (GIS©) [15] after 3 and 6 weeks of treatment. The GIS© comprises a validated questionnaire completed by the healthcare professional during a face-to-face interview with the patient [15]. It includes 10 items typically related to FD: epigastric pain/upper abdominal pain, abdominal cramps, bloating, early satiety, loss of appetite, sickness, nausea, vomiting, retrosternal discomfort and acidic eructation/heartburn. Intensity of each item is rated on a 5-point Likert scale (0 = no problem, 1 = mild problem, 2 = moderate problem, 3 = severe problem and 4 = very severe problem). With a maximum score of 4 points for each symptom, the questionnaire can yield a maximum score of 40, [15]. GIS© has been found to fulfil key criteria such as reproducibility, responsiveness to change and validity [15], and is suitable for evaluating the severity of FD and changes under treatment.

In addition, patients were asked for a subjective evaluation of the treatment effect at the end of treatment. The following score was used: 1 = very good, 2 = good, 3 = moderate, 4 = no effect. Where patients rated the efficacy on an intermediate level (e.g., 1.5), values were allocated to the next higher score for statistical analyses.

Safety and tolerability measures included recording of adverse events according to the Medical Dictionary for Regulatory Activities (MedDRA), as well as physical examination and measurements of vital signs.

### Statistical analysis

A sample size of 100 patients was planned for the study, although this was not based on a formal statistical power calculation.

Demographic data were described as means ± standard deviations. Missing data were not computed. The Wilcoxon rank sum test and Student’s t-test were both performed with the results of Wilcoxon rank sum test being used for tests with *N* < 40 and those of Student’s t-test when *N*≥40, with statistical significance at *p* ≤ 0.05. All analysis data sets and statistical outputs were produced using SAS version 9.3 (SAS Institute Inc., Cary, North Carolina, USA).

### Quality control (QC)

The study was monitored by an independent Clinical Monitor. The Clinical Monitor verified Informed Consent, safety-related data and completeness of the assessments. The biometrical report, integrated study report, and this publication were subject to additional QC measures.

## Results

### Patient demographics

A total of 97 patients were included in the study and treated with a fixed combination of pepsin and amino acid hydrochloride. A subgroup of 71 (74.2%) of these patients met the Rome III criteria for FD. Patients had a mean age of 58.4 ± 13.9 years and 63.2% were females, of whom 69.0% were postmenopausal. At baseline, 7.9% of the patients were smokers, 32.6% former smokers and 59.6% non-smokers; 60.9% of the patients occasionally consumed alcohol, 19.6% never, 15.2% once or twice per week and 4.4% daily. Almost all patients (96.8%) had good general health according to physicians’ judgements. Mean heart rate was 67.2 ± 6.8 min^−1^ and systolic and diastolic blood pressures 127.9 ±16.6 mmHg and 83.8 ± 10.0 mmHg, respectively.

Mean weight was 71.4 ± 13.7 kg, height 170.3 ± 8.5 cm. The majority (70.5%) reported constant weight during the 6 weeks preceding the study, while14.7% claimed gain (*n* = 5, 4.9 ± 1.6 kg) or loss (*n* = 8, 4.7 ± 2.1 kg).

Of the 97 patients included, 72 had GIS© score data for baseline and after 6 weeks of treatment. Two patients refused to take the prescribed medication and thus were not followed-up. Thirty-four patients had full sets of data at baseline, 3 and 6 weeks of treatment.

### Gastrointestinal symptom score

**Fig. 1:**
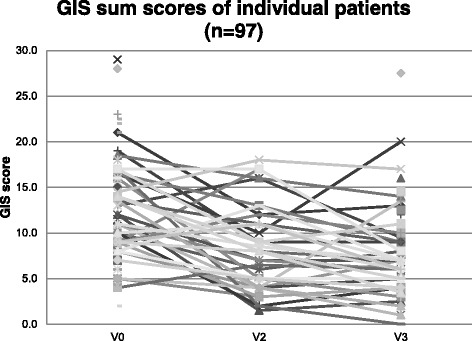
Comparison of individual GIS© sum scores (*n* = 97) at baseline, at the interim visit (21 ± 3 days), and at the end of treatment visit (42 ± 3 days) for patients with any available GIS© data

The overall GIS© sum score decreased from 11.6 before to 7.4 after a treatment period of 6 weeks, a decrease of 4.1 points (*p* < 0.0001; Table 2) reflecting an improvement of clinical symptomatology. For patients with GIS© data at 3 and 6 weeks, most of the decrease occurred during the first 3 weeks (Table 2). Changes in individual GIS© sum scores for all patients (*n* = 97) are shown in Fig. 1.

**Table 2:** Comparison of mean GIS© sum scores at baseline and after 6 weeks’ treatment

	N	Mean± Standard deviation (SD) (Minimum – Maximum)	Change from baseline
Patients with complete GIS© data for baseline and Week 6			
Baseline	72^a^	11.57 ± 4.8 (4–28)	─
Week 6	72^a^	7.43 ± 4.55 (0–27.5)	−4.14 (p < 0.0001^b^)
All patients with GIS© data at baseline and Week 3 and/or Week 6			
Baseline	94	11.47 ± 5.45 (2–29)	─
Week 3	37	8.36 ± 4.66 (1.5–18)	– 3.11 (*p* = 0.0002)
Week 6	73	7.51 ± 4.57 (0–27.5)	−3.96 (p < 0.0001)

^a^Number of patients with complete data for V0 and V3

^b^Statistically significant using Student’s t-test

Table 3 provides an overview of the individual GIS© symptom components at baseline and after 6 weeks. There were significant decreases in all single symptoms evaluated, except for nausea and vomiting.

**Table 3:** Specific Symptom scores of GIS© at baseline and after 6 weeks of treatment

Symptom	Score at baseline [Mean± SD (N)]	Score at 6 weeks [Mean± SD (N)]	P: Difference to baseline score
Epigastric pain/ upper abdominal pain	1.65 ± 1,04 (94)	1.12 ± 0,97 (73)	< 0.0001
Abdominal cramps	1.23 ± 1.18 (94)	0.74 ± 0.89 (72)	0.003
Bloating	2.12 ± 1.08 (94)	1.32 ± 1.08 (73)	< 0.0001
Early satiety	1.06 ± 1.00 (94)	0.81 ± 0.90 (72)	0.006
Loss of appetite	0.74 ± 1.01 (93)	0.44 ± 0.83 (73)	0.042
Sickness	0.98 ± 1.10 (94)	0.72 ± 0.95 (73)	0.017
Nausea	0.27 ± 0.74 (94)	0.15 ± 0.57 (73)	0.35
Vomiting	0.22 ± 0.72 (94)	0.10 ± 0.48 (73)	0.51
Retrosternal discomfort	1.40 ± 1.00 (94)	0.95 ± 0.88 (73)	0.019
Acidic eructation/ Heartburn	1.80 ± 1.12 (94)	1.20 ± 1.03 (73)	< 0.0001

GIS© data were available for 70 of the 71 patients who fulfilled the Rome III Criteria for FD. The mean baseline score in this subgroup was 11.1 (± 5.1), after 3 weeks this had decreased to 8.5 (± 4.6; *p* = 0.01) and after 6 weeks to 7.4 (±4.1; *p* < 0.0001). A GIS© score reduction of ≥50% was observed in 24.0% of patients (6 of 25 available questionnaires) after 3 weeks and in 30.8% (16 of 52 available questionnaires) after 6 weeks.

### Patients’ assessment of efficacy

Patients’ self-reported assessments of the effect of 6 weeks of treatment on their symptoms are shown in Table 4. Most (79.7%) rated their symptoms after treatment as very good, good or moderate.

**Table 4:** Patients’ self-reported assessments of medication efficacy at end of treatment

	Patient’s self-reported assessment (*N* = 74)			
	Very good [N (%)]	Good [N (%)]	Moderate [N (%)]	No effect [N (%)]
Number of entries	5 (6.76)	18 (24.3)	35 (47.3)	16 (21.6)

### Tolerability

There were 45 adverse events in 20 patients, for which either the physician or the study sponsor assumed a potential causality to the study medication. The vast majority of these were gastrointestinal symptoms, potentially also linked to the underlying FD. Five of these 20 patients did not change dosage, 1 patient reduced daily dose, 13 patients stopped the treatment, and 1 patient ceased medication intake due to presumed lack of efficacy. No serious adverse events were reported.

Other reasons for prematurely discontinuing study participation included lack of efficacy, leading to withdrawal by 6 patients (in addition to the 1 above), whilst another 5 withdrew from the study following freedom from symptoms, non-compliance with treatment, or with no reason given.

A comparison of vital signs documented at baseline and after 6 weeks of treatment showed no changes. Furthermore, there were no differences in abdominal girth measurements between baseline and at the end of treatment.

## Discussion

The aim of this observational study was to explore the effects of a fixed combination of pepsin and amino acid hydrochloride on symptoms in patients with FD. The primary outcome was change in GIS© with treatment. The GIS© sum score as well as the majority of its component scores were reduced significantly after 3 weeks treatment, with further changes observed after 6 weeks. There were no statistically significant score reductions for the GIS© vomiting and nausea items; however, few patients reported these two symptoms and the scores for these were also very low both before treatment initiation and after 6 weeks. In addition, in the subgroup of patients with symptoms meeting the Rome III criteria for FD, the reduction from baseline in GIS© was significant after 3 and 6 weeks.

Treatment tolerability in this study was good to moderate, with documented adverse reactions that were mostly related to gastrointestinal symptoms.

The patient population recruited for this non-interventional, observational study reflected the typical patient population with FD, with regard to age and gender [1, 5]. Patients with FD, and those included in this study, show a variety of different symptoms such as bloating, early satiety, epigastric pain, nausea, sickness and vomiting, retrosternal discomfort, heartburn, loss of appetite and abdominal pain, with these symptoms are present either alone or in combination [1–4]. A broad spectrum of factors have been implicated as causes of FD, ranging from *Helicobacter pylori* infection through inflammation to psychosocial issues [1, 2], and its management can be challenging once causes such as *Helicobacter pylori* have been excluded [3].

Validation and retrospective studies have previously shown that GIS© is a reliable and sensitive tool for the follow-up of FD-related symptoms [15, 16], enabling accurate differentiation of non-responders and responders. Furthermore, scores documented by gastroenterologists and general practitioners showed strong correlations, and a strong correlation to the symptom-specific components of the validated Nepean Dyspepsia Index has also been shown [15, 17]. With regards to interpretation of GIS©, a sum score of ≤10 corresponds to mild symptoms, where some symptoms may not be present at all and are thus impossible to improve [15, 16]. In this study, the mean baseline overall score was >10, but decreased to <10 for the whole study population as well as the large Rome III subgroup (74.2%). Placebo response in this field may affect up to 20% of patients [18, 19]; however, in this study, FD patients meeting the Rome III criteria, for example, improvements in GIS© were observed in about one third of patients after 6 weeks.

The fixed combination of pepsin and amino acid hydrochloride evaluated in the present study has traditionally used as a mild agent for support of gastric function in FD [8, 9]. Its precise mechanism of action is, however, unknown, although it is thought that the combination of pepsin and the acidic component should support digestion by aiding the stomach’s proteolytic activities [8, 9]. Further studies using in vitro or animal models could help shed light on the mechanism of action.

### Limitations

Firstly, this study had an observational design and was not a randomised clinical trial, the strength of the evidence obtained should therefore be weighted accordingly. Patients with acute or chronic FD symptoms were included and perhaps a larger study is warranted where these two groups can be evaluated separately. The study treatment was administered to individual patients at different dosages according to their treating physicians’ judgement. Therefore, although doses were within a dose range in the usage information [8], there may have been variations between patients in the dosages taken for a given level of symptoms. Finally, only 72 of 97 patients completed the study, with patients discontinuing mostly because of gastrointestinal symptoms, perceived as insufficient treatment and/or even possible worsening. Patients with a long history FD should be advised in future studies as well as in clinical practice, that their condition needs long term treatment as the beneficial effects may not become apparent for several weeks.

## Conclusions

The results of this observational study suggest that a fixed combination of pepsin and amino acid hydrochloride may be efficacious for the treatment of patients with FD. Good to moderate tolerability of this treatment is suggested. These findings should be further explored and evaluated in randomised controlled clinical trials.

## Abbreviations

FD: Functional dyspepsia
GIS©: Gastrointestinal symptom score
MedDRA: Medical dictionary for regulatory activities
QC: Quality control
